# Error-prone translation as a driver of proteostasis collapse and neurodegeneration

**DOI:** 10.4103/NRR.NRR-D-25-00795

**Published:** 2025-12-30

**Authors:** Rashid Akbergenov, David P. Wolfer, Dennis Gillingham, Dimitri Shcherbakov

**Affiliations:** 1 Biozentrum, University of Basel, Basel, Switzerland; 2 Anatomisches Institut, Universität Zürich, and Institut für Bewegungswissenschaften und Sport, ETH Zürich, Zurich, Switzerland; 3 Department of Chemistry, University of Basel, Basel, Switzerland

**Keywords:** aging, neurological disease, protein aggregation, protein misfolding, protein synthesis, proteostasis, ribosomal mistranslation, translation accuracy

## Abstract

Error-prone translation, resulting in inaccuracies in protein synthesis, is increasingly recognized as a critical contributor to proteostasis disruption and the pathogenesis of age-related neurological disorders. In recent years, numerous studies have elucidated that stochastic errors during mRNA translation may act as a molecular “tipping point” initiating pathogenic protein misfolding. A detailed analysis of how translation errors lead to protein misfolding, aggregation, and subsequent neurotoxicity will facilitate the identification of promising therapeutic targets for neurodegenerative diseases such as Alzheimer’s disease, Parkinson’s disease, and amyotrophic lateral sclerosis. This article explores the contribution of mistranslation to proteostasis decline, focusing on the unique vulnerabilities of neuronal cells. We review the sources of translation errors, effects of ribosomal ambiguity and error-restrictive mutations, role of proteostatic mechanisms (such as molecular chaperones, ubiquitin-proteasome system, and unfolded protein response), and provide a unified perspective that links age-related translational infidelity to neurodegeneration. By synthesizing the most recent data obtained with genetically modified cellular and animal model studies, we highlight how age-associated decline in translational fidelity exacerbates proteostasis failure and propose potential therapeutic interventions targeting translation accuracy to mitigate neurodegeneration.

## Proteostasis Imbalance and Protein Aggregation in Age-related Neurodegeneration

Age-related neurodegenerative diseases, such as Alzheimer’s disease (AD), Parkinson’s disease (PD), and Huntington’s disease (HD), share a common pathological hallmark: the accumulation of toxic, misfolded, and aggregated proteins. In AD, extracellular plaques composed of amyloid-β and intracellular neurofibrillary tangles formed by hyperphosphorylated tau protein are the key features. In PD, intracellular Lewy bodies rich in alpha-synuclein aggregates are characteristic, while HD is marked by aggregation-prone expanded polyglutamine tracts in the huntingtin protein. These misfolded, malfunctional, and aggregated protein species disrupt cellular homeostasis, interfere with essential cellular processes, and ultimately trigger apoptotic pathways, culminating in progressive neuronal loss.

The formation of such pathological protein aggregates is a direct consequence of disrupted protein homeostasis (proteostasis). Healthy proteostasis is the end result of a finely tuned network that regulates protein synthesis, folding, trafficking, maintenance, and degradation. Sustaining this balance is especially vital in neurons, which are largely post-mitotic. Neurons are also highly metabolically active, making them particularly vulnerable if proteostasis goes awry. Key players in the proteostatic network include the translation machinery, molecular chaperones such as heat shock proteins, intracellular trafficking systems, compartmentalized organelles such as the endoplasmic reticulum (ER), and proteolytic systems including the ubiquitin-proteasome system, lysosomes, and autophagosomes. Disruption in any of these systems can lead to proteostasis collapse and the accumulation of aggregation-prone proteins; documented examples include impaired autophagy in AD (Nixon, 2007), proteasome dysfunction in PD (McNaught et al., 2006), or chaperone system overload in HD (Muchowski and Wacker, 2005).

Historically, major efforts to understand and find a way to restore the mechanisms of proteostasis have focused primarily on the systems downstream of polypeptide biosynthesis, such as those responsible for protein folding, disaggregation, or degradation. These are molecular chaperones that are known to recognize and refold misfolded proteins or target them for degradation, while the ubiquitin-proteasome and autophagy-lysosome pathways act to clear proteins that are beyond repair. Thus, enhancing chaperone function has been shown to reduce polyglutamine aggregation in HD models (Lotz et al., 2010), and boosting autophagy can facilitate the clearance of toxic amyloid-β and tau species in AD models (Spilman et al., 2010).

Translation has always been understood as the factory for the production of new proteins, but recent efforts also reveal its critical importance for proteostasis. The fidelity and dynamics of protein synthesis can dramatically influence the fate of nascent polypeptides. Translation errors, ribosomal stalling, or imbalanced tRNA pools can lead to the production of structurally compromised proteins prone to misfolding and malfunction. Notably, studies have shown that aberrant translation is implicated in neurodegeneration: for instance, ribosomal pausing has been linked to tauopathy progression (Meier et al., 2016), and ribosomal stress has been observed in early stages of AD (Ding et al., 2005).

Moreover, translational control mechanisms, such as the integrated stress response (ISR), are increasingly recognized as critical modulators of proteostasis, in particular, in neuronal cells (Lockshin and Calakos, 2024). In models of PD, the short-term phosphorylation of α-subunit of the eukaryotic translation initiation factor 2 (eIF2α) (a key ISR event, reducing global translation) lowers the burden on the folding and degradation machinery and therefore is beneficial, but chronic activation can be deleterious (Colla et al., 2012). This duality underscores the fine balance in translation regulation required for maintaining proteostasis.

Thus, the paradigm is shifting from viewing translation as a passive participant to an active determinant of protein quality control.

## Link between Translation Fidelity and Proteostasis

**Figure 1:**
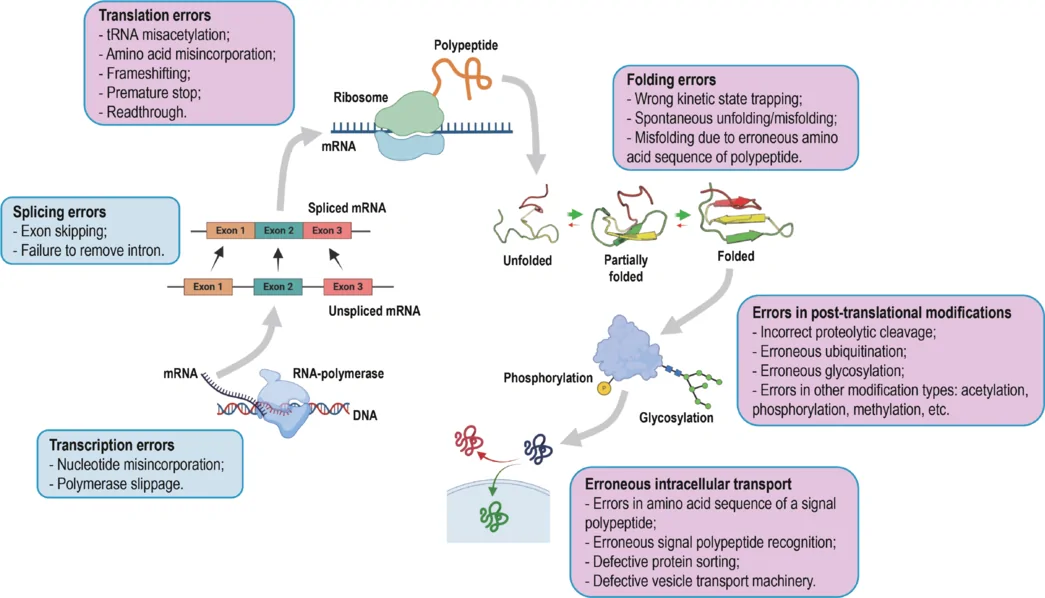
Sources of errors in eukaryotic protein biosynthesis and maturation. Errors may arise at many stages, from the transcription of genetic information to the folding, post-translational modification of the folded polypeptide, and intracellular transport. Stages where errors can be directly linked to mistranslation are highlighted with a magenta background. Adapted from Drummond and Wilke (2009) with BioRender.com.

Proteostatic pathways build an interconnected network comprising more than 2000 known components in mammals. Expression of genetic information into a fully functional protein comprises two major processes, protein biosynthesis and protein maturation, subdivided into several steps (**Figure 1**). Errors may arise at every step. Fidelity in mRNA translation, the least precise step of decoding of genetic information into polypeptide chain, is vital to proteostasis, while translation errors, though infrequent, potentially affect all downstream steps of protein maturation, producing misfolded and malfunctional or even toxic proteins that overwhelm the proteostasis network.

The intrinsic translational error rate is estimated to be rather low, between 10^-4^ and 10^-3^ per codon (Drummond and Wilke, 2009), yet even a modest increase of the error rate can potentially trigger proteotoxic stress responses, alter gene expression, reduce overall translation rates, and destabilize the genome (Paredes et al., 2012; Reverendo et al., 2014; Varanda et al., 2020).

Under normal conditions, molecular chaperones carefully monitor and facilitate protein folding (Gu et al., 2025). When the balance of protein homeostasis is disrupted (such as by elevated mistranslation or other stresses such as heat-shock), transcriptional programs dedicated to specific cellular reactions, such as the cytosolic stress response and/or the unfolded protein response (UPR) pathways, are activated in addition to the ubiquitin-proteasome system and autophagosome-lysosome pathway to aid refolding of misfolded proteins and to remove incurably misfolded and aggregated proteins (Higuchi-Sanabria et al., 2018).

The cytosolic stress response includes expression of heat shock proteins, primarily chaperones and disaggregases (such as Hsp104), activation of protein quality control systems and, at the later time point, formation of the stress granules – structures that form in the cytoplasm during proteotoxic stress, presumably sequestering misfolded/aggregated proteins (often together with chaperones or components of translation machinery) and preventing them from interfering with other cellular processes (Xu et al., 2023). The cytosolic stress response can also be triggered by mitochondrial stress, such as increased reactive oxygen species production or impaired mitochondrial function (D’Amico et al., 2017).

The UPR is a cellular stress response activated by the accumulation of misfolded or partially unfolded proteins in the ER. Signaling pathways of UPR are mediated by three ER-resident sensors: activating transcription factor 6 (ATF6), inositol-requiring enzyme 1 (IRE1), and protein kinase RNA-like ER kinase (PERK), controlled by ER-chaperone BiP. In the absence of ER proteotoxic stress, BiP binds to the ER luminal domain of the sensor proteins and inhibits their activation. However, accumulation of misfolded proteins in the ER lumen recruits BiP and induces its dissociation from the sensors, activating them. This accumulation, often a result of mistranslation, triggers a signaling pathway that aims to restore ER homeostasis by reducing protein synthesis (inhibition of translation), enhancing protein degradation (activation of ubiquitin-proteasome and autophagosome-lysosome systems), and increasing the ER’s folding capacity (upregulation of chaperones and other components of the folding machinery located in the ER). If the UPR fails to resolve the stress, it can lead to cellular apoptosis.

The ER is central to the proteostasis network, especially in managing secretory and membrane proteins.

Coupling between error-prone translation and UPR induction was demonstrated in many experiments. So, the cells expressing mutant tRNAs that misincorporate serine at leucine sites show increased protein ubiquitination and activate the ATF6 branch of the UPR, consistent with chronic ER stress (Varanda et al., 2020). Similar effects were observed in yeast and zebrafish models, where tRNA mutations-induced misincorporation of serine (Ser) at various non-cognate codon sites led to UPR activation and increased expression of heat shock proteins (Paredes et al., 2012; Reverendo et al., 2014). In human cell culture and mouse models, acute mistranslation-induced ER stress provoked by ribosome-binding aminoglycoside antibiotics was primarily mitigated by IRE1, initiating splicing of pro-UPR transcription factor XBP1. Resulted active form XBP1s initiated transcription of multiple UPR-associated genes, especially ER-chaperones (to refold potentially re-foldable misfolded proteins) and components of ERAD – ER-Associated Degradation pathway (to utilize incurably misfolded and aggregated proteins). Prolonged treatment with aminoglycosides resulted in chronic ER stress and ultimately cell death in spiral ganglion neurons (Oishi et al., 2015).

Another universal response to misfolded protein accumulation is attenuation of protein synthesis. Usually, it is initiated by PERK, a kinase that phosphorylates eIF2α, leading to the transient attenuation of protein synthesis (Kaufman, 2002). This modification limits the protein misfolding load by preventing the influx of newly synthesized proteins into the ER. In HEK293 cells expressing *ram* (ribosomal ambiguity) mutant protein RPS2 A226Y, increased mistranslation leads to protein aggregation, reduced translation, and upregulation of chaperone and proteasome genes (Shcherbakov et al., 2019). Notably, in HEK *RPS2*^*A226Y*^ cells translation attenuation occurs not at the initiation, but at the elongation step, possibly due to chaperone sequestration by misfolded proteins, which impedes ribosomal progression (Liu et al., 2013; Shalgi et al., 2013). While the mechanistic basis remains under investigation, the consensus is that mistranslating cells attenuate translation to alleviate proteotoxic load, whether through initiation or elongation control.

In contrast to acute mistranslation, chronic one leading to long-lasting overload with misfolded and non-functional proteins not only reduces translation and activates chaperones and the ubiquitin-proteasome system but also decreases ER protein translocation, thus shielding the ER from import of mistranslated proteins and preventing the ER folding machinery from an overload, which could trigger apoptosis through prolonged activation of the UPR stress sensors. Instead, the ER-destined proteins are rerouted to alternative pathways for proteolytic digestion, such as the mislocalized protein degradation pathway (Hessa et al., 2011), or trafficked to the Golgi for extracellular export (Shcherbakov et al., 2019).

Interestingly, chronic translation errors may even suppress classical ER UPR signaling and instead engage alternative cytosolic proteostasis mechanisms such as proteasome activation and stress granule formation, potentially as a tissue-protective adaptation (Moore et al., 2021).

In addition to partial silencing of the ER translocation and reduced activity of some UPR components, chronic mistranslation can lead to the transport of misfolded non-mitochondrial proteins into the mitochondria and related mitochondrial dysfunction. Those proteins can be actively imported to mitochondria for degradation, as was recently shown in yeast and human cell culture (Ruan et al., 2017; Shcherbakov et al., 2019), or the slowdown of protein import into the ER may promote mistargeting of proteins normally targeted to the ER to the mitochondria (Costa et al., 2018). Alternatively, misfolded abnormal proteins present in the ER can accumulate at ER-mitochondria contact regions, become associated with mitochondria, and even partially imported into the mitochondrial matrix, resulting in impaired mitochondrial function and activation of mitophagy as a protective mechanism (Li et al., 2019; Cortes Sanchon et al., 2021).

Thus, translation fidelity is intricately linked to proteostasis, influencing cellular health, stress responses, and disease progression. Translational errors, although generally rare, can overwhelm cellular quality control systems, triggering adaptive responses such as the cytosolic stress response and ER UPR. Internal and/or environmental perturbations that increase mistranslation not only compromise proteostasis but may also contribute to the etiology of aging and related neurodegenerative diseases, as we discuss below in more detail. Ultimately, maintaining high translational fidelity is not merely a question of efficiency but a cornerstone of cellular resilience and longevity.

## Cytosolic Translation: Brief Overview

Protein synthesis, or translation, is a fundamental cellular process in which ribosomes, supported by many other protein and non-protein factors, decode mRNA to produce functional proteins. This complex, multi-step process includes four general steps: initiation, elongation, termination, and ribosome recycling.

Cytosolic translation is very complex and carefully controlled through a sophisticated interplay of initiation factors and signaling pathways. It begins with initiation, which is generally the rate-limiting step of the process. Mature nuclear-encoded mRNAs undergo modifications such as a 5′-terminal 7-methylguanosine (m7G) cap and a 3′-poly(A) tail formation. These features are critical for ribosome recruitment and translation efficiency (Sachs et al., 1997; Michel et al., 2000).

Translation initiation is coordinated by two major eukaryotic initiation factor (eIF) assemblies: the eIF4F complex, which mediates mRNA recruitment, and the ternary complex, which delivers initiator tRNA to the 40S ribosomal subunit. The concerted action of these factors leads to the formation of the 48S pre-initiation complex. The 48S complex scans the 5′UTR of mRNA in a 5′ to 3′ direction until it encounters a start codon (AUG) and forms the stable 48S pre-initiation complex. The 60S ribosomal subunit then joins, mediated by eIF5B-guanosine triphosphate (GTP), and GTP hydrolysis prevents premature subunit dissociation (Acker and Lorsch, 2008). At this point, the ribosome becomes elongation-competent with the initiator tRNA in the P site and the next codon in the A site.

During elongation, aminoacyl-tRNAs (aa-tRNAs) bound to eEF1A-GTP are delivered to the ribosome. Selection depends on accurate codon-anticodon pairing in the A site. Non-cognate and most near-cognate tRNAs are rejected before GTP hydrolysis. Only cognate tRNAs (and occasionally near-cognate ones) are able to trigger conformational changes in the ribosome, constricting the decoding center and prompting GTP hydrolysis (Dever and Green, 2012). Incorrect tRNAs are rejected due to the high energy cost of mismatched pairing, while correct tRNAs lead to peptide bond formation after eEF1A (homolog of bacterial EF-Tu) release (Moazed and Noller, 1989). The ribosome then undergoes spontaneous rotation, with tRNAs shifting into hybrid states (A/P and P/E), which is stabilized by EF-G (or eEF2 in eukaryotes). The insertion of domain IV of EF-G into the decoding center initiates translocation, moving both mRNA and tRNAs and restoring the non-rotated state (Moazed and Noller, 1989; Ling and Ermolenko, 2016). Fidelity of the process in prokaryotes is enhanced by a secondary proofreading step in the P site after peptide bond formation (Ieong et al., 2016). Incorrect tRNAs can destabilize the ribosome and trigger translation termination. It was suggested that two-step proofreading mechanisms are at work not only in bacteria but also in eukaryotes and, perhaps, in all three kingdoms of life.

Elongation continues as the peptide chain grows, threading through the ribosomal exit tunnel and folding post-translationally (Cabrita et al., 2010). eIF5A factor may assist in elongation by enhancing the activity of eEF2 and promoting peptide bond formation (Schuller et al., 2017).

Translation terminates when the ribosome encounters a stop codon (UAA, UAG, or UGA) in the A site. The release factor eRF1, which structurally mimics tRNA, together with eRF3-GTP binds the stop codon in the A-site of the ribosome and triggers peptide release through GTP hydrolysis (Buckingham et al., 1997). The ribosome is then disassembled and recycled for new rounds of translation by the ABCE1–Pelota complex (Skabkin et al., 2013).

Tight regulation of the cytosolic mRNA translation allows rapid and reversible modulation of protein output in response to environmental, developmental, and stress-related cues (unlike transcription regulation, which is relatively slow and energetically costly). Translation regulation is especially prominent during cellular growth, differentiation, and response to nutrient availability or stress.

In mammals, the primary level of translation regulation occurs at translation initiation, where the activity of eIF4F complex is controlled by several signaling pathways, such as mTOR (mammalian target of rapamycin), which integrates nutrient and growth factor signals to modulate global protein synthesis. mTORC1 activity promotes cap-dependent translation by phosphorylating 4E-BPs (eIF4E-binding proteins), thereby releasing eIF4E to initiate translation (Proud, 2002). A parallel and stress-responsive pathway involves the phosphorylation of eIF2α, the alpha subunit of the translation initiation factor eIF2. Under normal conditions, eIF2 forms a ternary complex with GTP and initiator Met-tRNAi, essential for start codon recognition and initiation complex formation. However, upon cellular stress, such as ER stress, amino acid deprivation, viral infection, or oxidative stress, eIF2α becomes phosphorylated at Ser51 by one of four specific kinases: PERK, GCN2, PKR, or HRI. This phosphorylation event converts eIF2 into a competitive inhibitor of eIF2B, the guanine nucleotide exchange factor, effectively blocking translation initiation globally (Gray and Wickens, 1998).

## Mechanisms of Translation Fidelity and Infidelity in Eukaryotes

The fidelity of eukaryotic protein synthesis, being fundamental to cellular function and organismal viability, is governed by the interplay of several molecular systems, with critical control points at aminoacyl-tRNA synthesis, tRNA selection by the ribosome, and translocation events during elongation. Collectively, these mechanisms ensure that the vast majority of proteins are synthesized without errors, despite the inherent complexity of the translation process.

### Aminoacyl-tRNA synthetases and pre-ribosomal accuracy

The accurate formation of aminoacyl-tRNAs (aa-tRNAs) is the first checkpoint in translation fidelity. Aminoacyl-tRNA synthetases (aaRSs) are tasked with correctly pairing each tRNA with its cognate amino acid. These enzymes distinguish between 20 proteinogenic amino acids and structurally similar analogs using highly specific active sites. Nevertheless, due to chemical similarities among amino acids, improper amino acids can occasionally be misactivated. To mitigate this, aaRSs utilize pre-transfer and post-transfer editing mechanisms, either rejecting incorrect amino acids before tRNA charging or hydrolyzing them after tRNA charging. Some mischarged tRNAs can also be corrected in translation by stand-alone editing factors or the synthetase itself (Ling et al., 2009).

After accurate charging, the aa-tRNA forms a ternary complex with eukaryotic elongation factor 1A (eEF1A) and GTP. The bacterial ortholog, EF-Tu, has been shown to engage both the tRNA and amino acid, potentially recognizing and rejecting mis-aminoacylated tRNAs (Ling et al., 2009). Although the exact mechanism remains unresolved in eukaryotes, similar discriminatory principles are believed to apply.

### Ribosomal decoding and kinetic proofreading

In the context of the genetic code and protein biosynthesis, cognate codons are those that perfectly match an aminoacyl-tRNA (aa-tRNA) anticodon and allow for the correct incorporation of amino acids into a growing polypeptide chain. Near-cognate codons have a single nucleotide mismatch with an aa-tRNA anticodons, allowing for some interaction and potentially leading to amino acid misincorporation. Non-cognate codons with more than one mismatch have no or very little interaction with an aa-tRNA and are not involved in translation. The majority of translational errors, or mistranslation, involves the misincorporation of amino acids at near-cognate codons by the ribosome due to errors in codon recognition or at non-cognate codons due to errors in aminoacyl-tRNA synthesis (Mohler and Ibba, 2017).

Decoding fidelity is maintained by a two-step selection process on the ribosome. Upon delivery of the ternary complex to the ribosome’s A site, the mRNA codon is “sampled” for correct base pairing with the tRNA anticodon. Non-cognate and most near-cognate complexes are rejected at this step before GTP hydrolysis. Cognate codon-anticodon interaction triggers conformational changes in the 40S subunit, “closing” the decoding center and promoting GTP hydrolysis by eEF1A. This results in the release of eEF1A-GDP and accommodation of the aa-tRNA into the peptidyl transferase center (Rozov et al., 2016; Prabhakar et al., 2017).

Following aa-tRNA accommodation, a secondary proofreading step occurs before peptide bond formation. This kinetic proofreading exploits subtle differences in binding energies between correct and incorrect codon-anticodon pairs, enabling discrimination even among near-cognate tRNAs. After peptide bond formation, tRNAs adopt a hybrid state, with anticodons in the A and P sites and acceptor stems in the P and E sites. Translocation of the ribosome, catalyzed by eukaryotic elongation factor 2 (eEF2), restores the ribosome to the classical state and ensures maintenance of the reading frame.

### Error frequencies and codon-anticodon mismatches

There are four major types of translation errors: (1) Amino acid misincorporation occurs when an incorrect amino acid is incorporated into a growing polypeptide chain as a result of mischarged tRNAs or codon-anticodon mismatches during mRNA decoding. (2) Stop codon readthrough, which involves translating ribosome ignoring stop codon and producing extended proteins. This can be due to near-cognate tRNAs recognizing stop codons or modifications in mRNA structure. (3) Ribosomal frameshifting occurs when the ribosome shifts reading frame, producing alternative polypeptides (often very short and non-functional). Frameshifting can be programmed or result from slippery sequences and/or distinctive secondary mRNA structures. (4) Premature translation termination occurs when an amino acid-coding triplet is recognized as a stop-codon, often leading to truncated and non-functional protein.

Translational errors are rare but not uniform. In particular, missense errors arising either from mischarged tRNAs or incorrect codon recognition have been reported to have error rates ranging from 10^-5^ to 10^-3^. Stop-codon readthrough typically occurs at a frequency of less than 10^-3^ varying between codons (UAA, UAG, or UGA). However, in some cases, the readthrough level can be significantly higher, exceeding 10^-2^. The specific frequency is influenced by the nucleotide context surrounding the stop codon, in particular, the 3’-UTR.

Chemical modifications in tRNA anticodons and mRNA nucleotides also contribute to translational ambiguity (Rozov et al., 2016; Suzuki, 2021). These modifications can mimic canonical base pairing or alter decoding center dynamics. Despite this, the distribution of translation errors is nonrandom. Certain proteins and sequence regions are more prone to errors, possibly reflecting differences in codon context, mRNA structure, or local translation dynamics.

Recently, advanced proteomic techniques, particularly mass spectrometry, have revealed codon-specific error patterns in model organisms such as *E. coli* and yeast, providing a nuanced view of translation errors beyond classical gain-of-function reporter assays (Mordret et al., 2019). These studies show that translational error rates can spike in response to stress, genetic mutations, or disease states. For instance, human colon cancers exhibit elevated translational errors due to aberrant tRNA expression (Santos et al., 2018).

### Modulators of translational fidelity

Translation efficiency is inversely correlated with error rates: naturally highly expressed genes are translated with greater fidelity, whereas forced overexpression can increase error frequency (Garofalo et al., 2019; Mordret et al., 2019).

In the eukaryotic cytosol, translational fidelity is subject to modulation by a variety of molecular and environmental cues. The sequence context of the mRNA, including features such as secondary structures, upstream open reading frames, and codon usage bias, plays a pivotal role in regulating ribosomal scanning and the accuracy of start codon recognition. These contextual elements can influence both the rate and precision of translation initiation, potentially leading to downstream effects on elongation fidelity. Furthermore, the availability and relative abundance of tRNA species within the cytosol critically impact decoding accuracy. Imbalances in tRNA pools, whether due to differential gene expression, cellular stress, or exogenous perturbations, can lead to the misreading of codons and elevated rates of amino acid misincorporation, particularly under conditions where the availability of certain cognate tRNAs is limited (Mordret et al., 2019).

In addition, post-transcriptional mRNA modifications, such as N1-methylpseudouridine, introduced naturally or through therapeutic engineering, have been shown to enhance mRNA stability and translation rates. However, these modifications can also perturb ribosome dynamics and promote frameshifting or miscoding under some ORF contexts (Rozov et al., 2016; Ranjan and Leidel, 2019). Cellular stress, including oxidative damage, amino acid starvation, or heat shock, further destabilizes translational fidelity by altering ribosome function and elongation dynamics (Wohlgemuth et al., 2021).

## Age-associated Decline in Translational Fidelity Contributes to Loss of Proteostasis with Aging

The maintenance of proteostasis constitutes a formidable cellular challenge, as most proteins are only boundedly stable under physiological conditions and are persistently susceptible to misfolding. To preserve the integrity of the proteome, cells employ a sophisticated proteostasis network (PN), which facilitates the correct folding of nascent polypeptides, recognizing and refolding of damaged proteins, and targeting irreversibly misfolded species for degradation.

### Components of the cellular proteostasis network

The PN includes molecular chaperones, folding enzymes (performing posttranslational modifications), the ubiquitin-proteasome system, autophagy-lysosome pathways, and stress response pathways such as the unfolded protein response of the endoplasmic reticulum (UPR^ER^) and mitochondria (UPR^mt^) (Labbadia and Morimoto, 2015). In young organisms, these systems operate at high efficiency, dynamically adjusting the cellular proteome according to environmental and physiological stresses. However, during aging, multiple components of the PN undergo a functional decline: (1) Molecular chaperones exhibit reduced expression and activity, impairing nascent protein folding and refolding of stress-denatured proteins (Powers and Balch, 2008). Chaperone gene expression becomes epigenetically repressed with age, limiting the cellular capacity to buffer proteotoxic stress (Wang et al., 2022). (2) Ubiquitin-proteasome system activity diminishes with age, leading to decreased degradation of ubiquitinated proteins and accumulation of damaged proteins (Margulis et al., 2020), whereas non-specific and potentially harmful post-translational modifications such as oxidation, glycation, and carbamylation become more frequent, destabilizing protein structure and functions (Dukan et al., 2000; Nicolas et al., 2019). Especially harmful is oxidative damage of the proteasomal subunits, which reduces the proteolytic efficiency and contributes to the further accumulation of defective polypeptides (Chondrogianni and Gonos, 2008). (3) Autophagic flux declines, impairing the clearance of aggregated proteins and dysfunctional organelles, especially mitochondria (Wong et al., 2020). (4) Stress responses, such as UPR^ER^ and UPR^mt^, show reduced activation thresholds and resolution capacity, leading to chronic stress states exacerbating cellular dysfunction (Taylor and Dillin, 2011).

Among the multiple molecular mechanisms that have been identified as driving the age-associated decline in proteostatic capacity, the key ones are translation related: decline in translational fidelity, leading to an increased burden of misfolded and malfunctional proteins entering the PN (Mordret et al., 2019; Bottger et al., 2025) and increased ribosome pausing and ribosome collisions driving abortive polypeptide synthesis, nascent polypeptide aggregation, and overload of the less efficient ribosome-associated quality control pathway (Filbeck et al., 2022; Stein et al., 2022).

### Role of translation fidelity in proteostasis network maintenance

Recent studies have highlighted mRNA translation as an emerging constraint on protein homeostasis during aging. Evidence suggests that age-associated changes in cellular mechanisms increasingly impair ribosome function, manifesting as decreased translation elongation rates, a decoupling of transcription and translation processes, and disruptions in ribosome stoichiometry driven by a decline in proteasome activity (Anisimova et al., 2020; Kelmer Sacramento et al., 2020; Gerashchenko et al., 2021), reviewed in (Llewellyn et al., 2024). Among these, a critical role has been attributed to translational fidelity, as comparative analyses across rodent species revealed a positive correlation between translational accuracy and maximum lifespan, suggesting that higher fidelity in protein synthesis may confer resistance to the deleterious effects of aging (Ke et al., 2017).

The notion that translation errors contribute to aging has a long-standing history, initially proposed by the error catastrophe theories of the 1960s (Orgel, 1963, 1970; Edelmann and Gallant, 1977). Early investigations during the 1980s sought to determine whether translational fidelity declines with organismal or cellular aging. These studies, however, generally failed to detect significant increases in translation errors with age (Anisimova et al., 2018; Ke et al., 2018), leading to the prevailing belief that translation fidelity remains largely unaffected throughout life. Yet, these early assays were limited by poor sensitivity, as available methods were inadequate to detect the inherently rare events of amino acid misincorporation (Luce and Bunn, 1989; Rattan, 1996).

**Figure 2:**
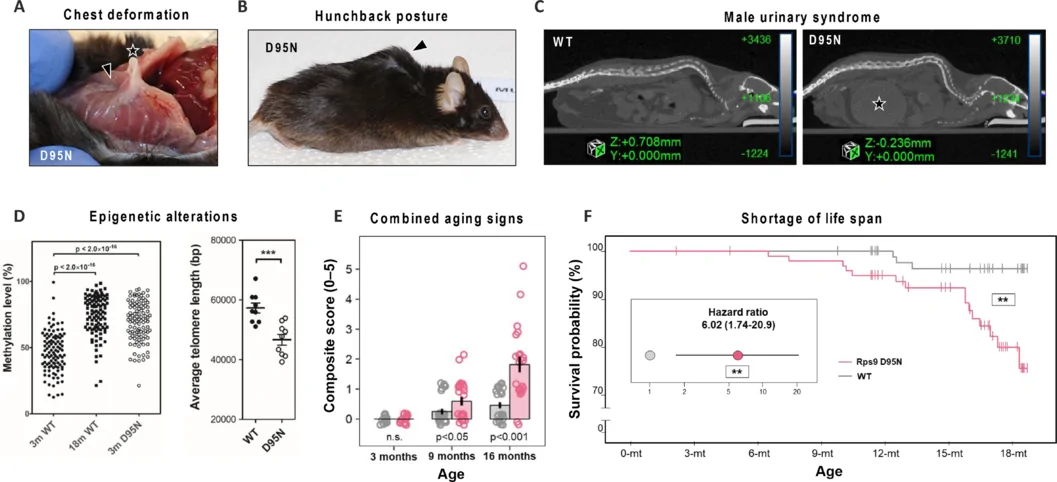
Premature aging phenotypes associated with error-prone translation in transgenic mice carrying the RPS9 D95N mutation. (A) Chest deformities observed at the age of 16 months: sternum (arrowhead) with protruding xiphoid (star). (B) Typical hunchback posture at the age of 16 months. (C) Mouse urinary syndrome: micro-CT images of a 17-month male mutant with error-prone translation (right) and a 17-month male wild-type (WT) mouse (left). The mutant mouse shows a massively filled bladder (star), while the bladder is empty and thus invisible in the WT mouse. (D) Epigenetic alterations in mutant mice in comparison to WT mice. Left: methylation levels (in %) of 104 age-related CpG sites in 3-month-old WT, 18-month-old WT, and 3-month-old mutants (*n* = 4 for each group, each point represents the mean). Right: length of telomeres in the brain of 3-month-old WT and mutant mice (*n* = 9; ± SEM). ^***^P < 0.001. (E) Composite score reflecting the presence or absence of five aging signs: chest deformation, hunchback posture, altered fur (alopecia, depigmentation, and scruffiness), eye problems (cataract and inflammation), and poor body condition. Rosa, mutants; gray, WT. Mean, SE, and individual data points plotted with vertical and horizontal jittering. ^***^P < 0.001, ^*^P < 0.05, ^n.s.^*P* ≥ 0.1. (F) Effect of the ribosomal error-prone mutation on survival (Kaplan-Meier: *P* = 0.0017) and hazard ratio of mutant versus WT mice (inset, Cox proportional hazards model: *P* = 0.005). Rosa: mutants; gray: WT. Reprinted with permission from Shcherbakov et al. (2022).

Contemporary research has substantially reshaped this perspective. More recent studies (**Figure 2**) emphasize translational fidelity as a crucial determinant of aging, suggesting that translational fidelity coevolves with longevity in mammals, and that artificially increased ribosomal error rates result in premature aging phenotypes and shortened lifespan (Ke et al., 2017; Moore et al., 2021; Brilkova et al., 2022; Shcherbakov et al., 2022). Moreover, it has been shown that whereas translational errors, such as stop-codon readthrough, increase during aging, indicating a progressive decline in translation accuracy over time in *Drosophila melanogaster*, enhancing translational fidelity with a genome-wide error-restrictive mutation *RPS23*^*K60R*^ has been demonstrated to extend lifespan in yeast and non-mammalian metazoans *D. melanogaster* and *Caenorhabditis elegans* (Martinez-Miguel et al., 2021). Conversely, knock-in mice harboring the *RPS9*^*D95N*^ ribosomal ambiguity (ram) mutation, which increases ribosomal error rates, exhibit premature aging phenotypes and reduced lifespan, providing direct evidence for the causal role of translational infidelity in mammalian aging (Shcherbakov et al., 2022).

**Figure 3:**
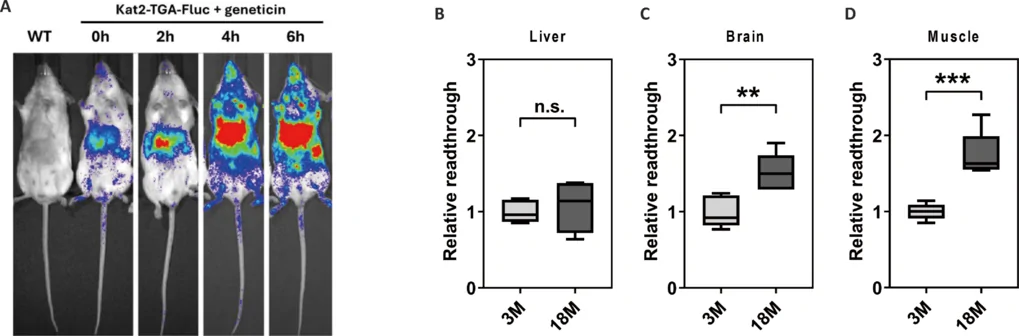
*In vivo* system for measuring mistranslation and observed age-associated increase in mistranslation. (A) *In vivo* induction of translation errors in mice by injection of geneticin, a well-established inducer of mistranslation in mammals. The transgenic mice carry a reporter cassette (Kat2-TGA-Fluc), where Firefly luciferase (Fluc) expression is dependent on translational readthrough of a premature stop codon (TGA). Increased Fluc activity reflects elevated levels of mistranslation. (B–D) Age-associated increase in relative translation readthrough as detected in tissue extracts from (B) liver (C) brain, (D) muscle of mice at the ages of 3 and 18 months (M). Reprinted with permission from Böttger (2025).

Finally, a tissue-dependent increase in mistranslation rates with age has been directly demonstrated *in vivo* in mice (**Figure 3**), highlighting the age-related decline in translational fidelity as a potential contributor to the aging process. (Bottger et al., 2025).

Thus, age gradually increases the inherent error predisposition of protein biosynthesis, producing a continual and rising flux of destabilized and misfolded proteins. Amino acid misincorporation due to codon-anticodon mismatches, while infrequent and stochastic, remains the principal limitation to the accuracy of protein biosynthesis and becomes more frequent. Beyond promoting misfolding, translational errors compromise proteome integrity through additional mechanisms, including increased susceptibility to oxidative damage (Dukan et al., 2000; Krisko and Radman, 2013), thereby exacerbating proteotoxic stress and contributing to the gradual collapse of proteostasis (Drummond and Wilke, 2008; Powers and Balch, 2008).

### Translation fidelity and aging

Aging, broadly understood as a progressive systems-level pathology, is characterized by subtle yet cumulative impairments across multiple cellular processes (López-Otin et al., 2023). Experimental models with induced elevations in translation error frequency exhibit pleiotropic manifestations that mirror many hallmarks of aging, including increased protein aggregation, telomere shortening, altered DNA methylation patterns, mitochondrial dysfunction, elevated reactive oxygen species production, lipid peroxidation, and oxidative protein damage (Shcherbakov et al., 2019, 2022; Brilkova et al., 2022). Notably, the introduction of a single ribosomal ambiguity mutation (*RPS9*^*D95R*^) suffices to recapitulate a broad spectrum of biochemical, cellular, and physiological features associated with natural aging.

These findings align with the view that, in addition to the intrinsic error-prone nature of translation and the well-documented age-related decline in proteostasis capacity (Hipp et al., 2019), an age-associated increase in ribosomal error rates contributes significantly to the aging process. Interestingly, this increase in translational infidelity has been observed primarily in post-mitotic tissues, such as skeletal muscle and brain, where proteostasis maintenance is particularly critical. In murine models, elevated ribosomal error rates emerge during late middle age, a stage that coincides with the onset of measurable physiological and behavioral declines (Prenderville et al., 2015; Brilkova et al., 2022; Clayton et al., 2022; Shcherbakov et al., 2022; Murray et al., 2024), further supporting the hypothesis that loss of translational fidelity is a driving factor in age-related deterioration.

It is important to note that the vast majority of recent studies accurately measuring translation fidelity and linking its decline to shorter lifespan and age-related disorders have been conducted in invertebrates (Martinez-Miguel et al., 2021) or, at best, in rodents (Ke et al., 2017; Shcherbakov et al., 2022; Böttger et al., 2025). There are only a few publications that have measured translation fidelity in human tissues or primary cell cultures using modern, sensitive assays (see the next chapter). Given that the human lifespan exceeds that of mice by approximately twenty-fold, it is possible that humans have evolved more robust mechanisms to maintain translational fidelity, as has been observed in long-lived rodent species (Ke et al., 2017). Further research is needed to clarify the role of translation fidelity, specifically in human aging and age-associated diseases.

However, based on available data, we can postulate that age-related decline in translational fidelity and associated loss in proteostasis capacity represents a central hallmark of aging in eukaryotes. Errors in translation together with other alterations in codon usage, tRNA dynamics, translation initiation and elongation rates, and quality control responses synergistically promote the accumulation of misfolded, malfunctional, and aggregated proteins, driving cellular dysfunction and disease susceptibility.

It will be a challenge for future studies to elucidate the exact factors that mediate the age-related decline in translation fidelity and to pinpoint the molecular mechanisms and signal transduction pathways that convert errors in protein synthesis into aging and age-related disease, with the view to developing potential therapeutic targets for the benefit of healthy aging and amelioration of age-related diseases.

## From Translational Errors to Proteotoxicity and Neurodegenerative Diseases

Aging is the major risk factor for the development of neurodegenerative diseases (NDDs), including AD, PD, amyotrophic lateral sclerosis, and HD. A hallmark of all these conditions is the progressive accumulation of misfolded, aggregation-prone proteins in affected brain regions (Wilson et al., 2023). While various processes contribute to this proteotoxic stress, a decline in mRNA translation fidelity during aging seems to be one of the critical upstream factors that compromise proteostasis and promote neurodegeneration.

### Link between translation fidelity and harmful protein aggregation

Once the aged and less active PN in neuronal cells becomes overloaded with newly synthesized mistranslated and misfolded proteins, those proteins start to accumulate and coalesce into aggregates, some of which serve as seeds for further aggregation. Such aggregates are a central feature of NDDs: amyloid-β and tau in AD, α-synuclein in PD, polyglutamine-expanded huntingtin in HD, and TDP-43 in amyotrophic lateral sclerosis (Chiti and Dobson, 2017; Boeynaems et al., 2018). Notably, these aggregates often sequester molecular chaperones and other components of the PN, unsuccessfully trying to resolve the newly formed aggregates, exacerbating cellular stress and establishing a feed-forward cycle of proteotoxicity. Moreover, aggregated proteins can sequester translation-associated ribosome-associated quality control components, reducing their availability and exacerbating mistranslation-induced toxicity. Normally, the ribosome-associated quality control system detects and resolves stalled translation events and aberrant nascent chains; however, age-related impairments in this pathway, caused by co-aggregation of its components, compromise co-translational protein quality control and are increasingly recognized as contributors to neurodegeneration (Wang et al., 2025).

Traditional models of NDD development have focused heavily on the accumulation of protein aggregates such as amyloid-β, tau, or α-synuclein. However, these models often fail to explain why such aggregates only become pathogenic late in life or why similar levels of aggregation (detected *post-mortem*) can occur without disease. A growing body of research supports a more nuanced “Two-Hit” model, wherein age-related proteostasis decline constitutes the “first hit”, and a loss of translational fidelity serves as a “second hit” that synergistically triggers neuronal degeneration. Aging cells, especially highly vulnerable post-mitotic neurons, experience a gradual decline in proteostasis capacity. This includes reduced expression and activity of molecular chaperones, diminished proteasome efficiency, and impaired autophagic clearance of misfolded proteins and aggregates (Gandhi et al., 2019). These changes create a quasi-stable proteotoxic environment where even modest amounts of aberrant proteins can overwhelm clearance systems and accumulate into progressing oligomeric or aggregated forms. While aging alone does not necessarily lead to neurodegeneration, it sets the stage by narrowing the cellular tolerance for proteostatic disruptions. A factor reducing ribosomal accuracy and resulting in widespread mistranslation and increased production of misfolded proteins would create the “second hit” affecting cellular proteostasis. Thus, translation errors act as a “tipping point” when combined with age-related proteostasis deficits, explaining why neurodegeneration emerges only when both insults converge (Iben, 2023; Rochet, 2023).

One major source of mistranslation arises from defects in aaRSs, enzymes responsible for charging tRNAs with their cognate amino acids. Under normal conditions, aaRSs maintain high fidelity through pre- and post-transfer editing mechanisms; however, the process of precise tRNA aminoacylation can be affected by internal or external stress factors. Genetic studies in animal models have shown that even modest increases in tRNA mischarging can lead to neurodegeneration. For example, a mutation in the editing domain of alanyl-tRNA synthetase (AARS) in mice doubles the baseline level of mischarging of tRNAAla with serine, leading to progressive degeneration of Purkinje cells (Lee et al., 2006). More severe editing defects in AARS result in broader neurological phenotypes, underscoring the vulnerability of neurons to mistranslation (Liu et al., 2014). Similarly, in *D. melanogaster*, mutations in phenylalanyl-tRNA synthetase that increase mischarging of tRNA^Phe^ with tyrosine induce neurodegeneration (Liu et al., 2014). These mutations consistently lead to the upregulation of molecular chaperones, accumulation of misfolded protein aggregates, and activation of the UPR, indicating a chronic overload of the PN.

In humans, several dominant and recessive mutations in cytoplasmic aaRS genes have been identified in patients with neurological disorders. Notably, heterozygous mutations in AARS are associated with syndromes such as microcephaly, hypomyelination, and epilepsy (Nakayama et al., 2017). One such allele severely compromises the editing function of AARS *in vitro*, reducing its ability to hydrolyze Ser-tRNA^Ala^ and increasing aaRS-induced mistranslation. However, the same mutation also slightly reduces overall aminoacylation activity, thus complicating the interpretation of its direct impact on translation fidelity and disease progression.

**Figure 4:**
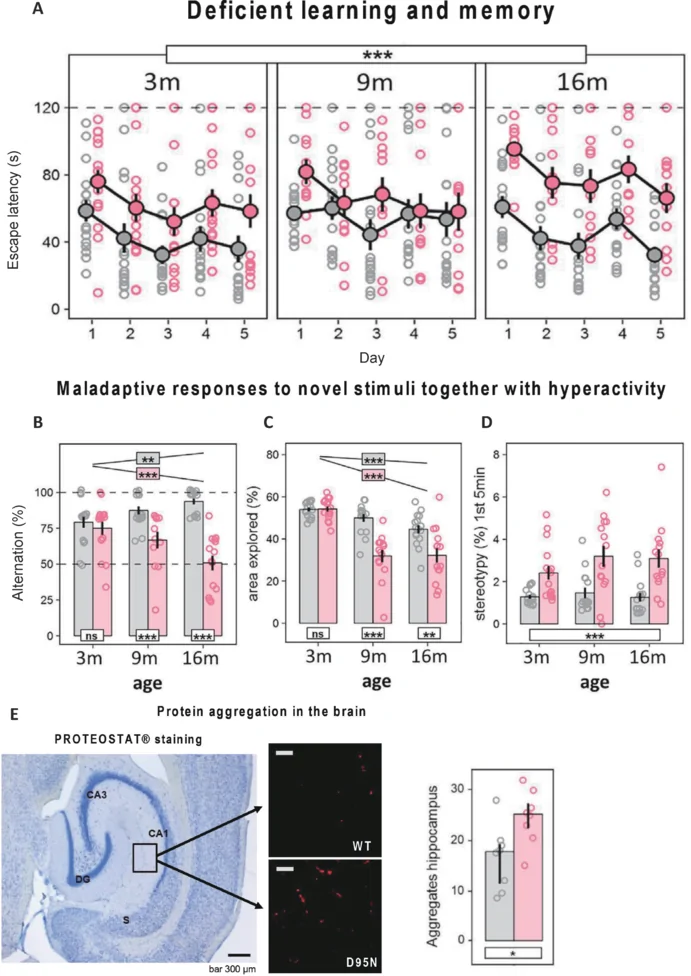
Signs of neurological disorder in transgenic mice carrying the RPS9 D95N mutation, known to reduce translational fidelity. (A–D) Graphs show untransformed mean, SE, and individual data points. Mutant animals are represented in rosa and wild-type (WT) controls in gray; ^*^P < 0.05, ^**^P < 0.01, ^***^P < 0.001, ^ns^*P* ≥ 0.1. (A) Deficient learning and memory as assessed by escape latency during training in the water maze place navigation task as function of day (average of six trials). (B–D) Maladaptive responses to novel stimuli together with hyperactivity as assessed by: (B) fraction of trials with alternating choices in the T-maze spontaneous alternation test; (C) Average fraction of large open field arena surface explored; (D) Stereotypy index (fraction of path consisting of repeated movement patterns) during the first 5 minutes spent in the large open field. (E) Nissl-stained section of the hippocampal formation (DG dentate gyrus, CA1 hippocampal subfield CA1, CA3 hippocampal subfield CA3, S subiculum) and PROTEOSTAT protein aggregation assay in brain hippocampal C1 sections of 18-month-old mice. ns: Not significant. Reprinted with permission from Brilkova et al. (2022).

Alongside aaRS defects, ribosomal mutations have also been implicated in translational fidelity loss and neurodevelopmental pathology. The role of ribosome in decoding accuracy is critical, and mutations that increase error rates can have severe outcomes. Transgenic mice harboring the *RPS9* D95N ribosomal ambiguity (*ram*) mutation in protein uS4 (**Figure 4**) show increased translation error rates, resulting in premature aging, reduced lifespan, and neurobehavioral deficits reminiscent of early AD-like symptoms (Brilkova et al., 2022; Shcherbakov et al., 2022). Conversely, models engineered for improved translational fidelity with the *RPS23* K60R mutation in ribosomal protein uS12, involved in stabilizing the ribosome decoding center, display extended lifespan and enhanced health-span in yeast, *C. elegans*, and *Drosophila* (Martinez-Miguel et al., 2021).

### Some human pathogenic mutations are associated with a decline in translation fidelity

Human genetic data further support these observations. Some missense mutations in *RPS23*, which encodes the ribosomal protein uS12, are linked to microcephaly, hearing loss, and intellectual disability (Paolini et al., 2017). In particular, the R67K mutation reduces translational fidelity and increases frameshifting and suppression of nonsense mutations in the yeast model. Patient-derived fibroblasts carrying this mutation exhibit increased stop codon readthrough, confirming a defect in translation fidelity. The R67K substitution disrupts post-translational hydroxylation of a neighboring proline by OGFOD1, an oxygenase that modulates decoding accuracy and stop codon recognition (Loenarz et al., 2014; Singleton et al., 2014). These *RPS23* R67K mutant ribosomes are underrepresented in polysomes, suggesting partial exclusion from active translation and showing that even low levels of erroneous translation can have profound developmental consequences.

Interestingly, experiments with heterozygous *RPS23*^*R67K/+*^ *D. melanogaster* did not reveal decreased translation fidelity in transgenic animals compared to wild-type controls (Recasens-Alvarez et al., 2021). Therefore, the authors speculated that the observed proteotoxic stress in *RPS23*-deficient tissues may result from an accumulation of unassembled ribosomal proteins, thereby overloading cellular protein degradation pathways. Consistent with this model, inhibition of TOR activity, which suppresses ribosomal protein synthesis, reduced overall translation rates and stimulated autophagy, ultimately mitigating proteotoxic stress.

Additional evidence implicates ribosomal protein *RPL10* (uL16) in translational fidelity and neurodevelopment. Missense mutations in *RPL10* are associated with autism, X-linked intellectual disability, and cerebellar hypoplasia (Brooks et al., 2014; Thevenon et al., 2015; Zanni et al., 2015). While some patient mutations alter ribosome profiles or affect translational output, the precise fidelity defects remain unresolved. Nevertheless, studies in yeast indicate that *RPL10* mutations can impair translational accuracy (Sulima et al., 2014), implying that mistranslation may be involved in the disease phenotypes in affected individuals.

Non-ribosomal and not directly translation-associated factors can also affect translation fidelity, leading to severe inherited genetic syndromes that present with symptoms resembling age-related disorders. In trichothiodystrophy (TTD), an autosomal recessive neurodevelopmental disorder characterized by intellectual disability, sulfur-deficient brittle hair, ichthyosis of the skin, and signs of premature aging, pathogenic mutations can affect RNA polymerase I elongation and the DNA-repair factor TFIIH, the RNA polymerase II transcription initiation factor TFIIEβ, certain tRNA synthetases, the *C7orf11* gene product TTDN1, or the splicing factor RNF113A (Zhu et al., 2023). In Cockayne syndrome, an autosomal recessive disorder marked by developmental delay, multi-organ degeneration, and premature aging, pathogenic mutations occur in genes encoding five different DNA repair proteins (CSA, CSB, XPB, XPD, and XPG) of the nucleotide excision repair pathway (Alupei et al., 2018). Although the genes involved in the pathogenesis of these two disorders differ, they share some common features: they participate in rRNA turnover and ribosomal biogenesis. For example, the CSA and CSB genes play important roles in RNA polymerase I activity (Qiang et al., 2021). Consequently, certain pathogenic mutations in these genes can induce translation infidelity, provoke protein misfolding, and cause a general loss of proteostasis (Alupei et al., 2018; Khalid et al., 2023). Moreover, a recent study by Wagner et al. (2024) links ribosomal mistranslation and the resulting proteome instability to the age-related neurodegenerative Huntington’s disease, further highlighting the connection between translation fidelity, proteostasis, and neuronal health.

Collectively, these findings underscore the importance of accurate mRNA translation in maintaining neuronal proteostasis. Both aaRS and ribosomal protein mutations that compromise translation fidelity lead to protein misfolding, aggregation, and activation of stress responses. Given the limited regenerative capacity of neurons, such cumulative effects are particularly detrimental and may serve as an initiating factor in the pathogenesis of age-related NDDs. As translation fidelity declines with age, the burden of mistranslated proteins may overwhelm the PN, contributing to neurodegenerative cascades and the progression of disease.

## Promising Directions for Further Research and Therapy Development

Many recent analyses implicate decreased translational fidelity and associated overload of protein degradation machinery as cardinal events in the general loss of proteostasis. These proteostatic disruptions contribute to (and perhaps dictate) what we see as “normal” aging, as well as its gradual progression into numerous age-related neurological and neurodegenerative disorders. Therefore, enhancing translational accuracy through genetic or pharmacological interventions represents a promising avenue for delaying or mitigating age-associated neurodegenerative disorders.

### Potential of gene therapy

One approach utilizes error-restrictive mutations. Ribosomal proteins, particularly those located in or near the decoding center of the ribosome, participate in accurate tRNA selection and codon recognition. As such, mutations in these proteins can alter the ribosome’s fidelity. Ribosomal ambiguity (ram) mutations reduce the stringency of decoding, increasing mistranslation rates. Such mutations, e.g., *RPS9* D95N in mice, lead to widespread proteome instability, protein aggregation, premature aging, and cognitive deficits, thus resembling early-stage AD pathology (Brilkova et al., 2022; Shcherbakov et al., 2022). In contrast, error-restrictive mutations in ribosomal proteins make decoding more stringent and improve translational fidelity. One notable example is the K60R mutation in *RPS23*, encoding ribosomal protein uS12, which enhances the accuracy of codon-anticodon pairing without significantly compromising translation efficacy (Martinez-Miguel et al., 2021). This mutation stabilizes the ribosome conformation upon codon recognition, which reduces the acceptance of near-cognate tRNAs and suppresses stop codon readthrough and frameshifting errors. In eukaryotic model organisms *Saccharomyces pombe*, *C. elegans*, and *D. melanogaster*, this mutation assures increased lifespan, delayed proteostasis deterioration, and enhanced stress resilience.

Those protective effects of error-restrictive ribosomes suggest a potential to prevent or mitigate NDDs. Enhancing translation accuracy could theoretically limit the generation of aggregation-prone proteins, reduce proteotoxic stress, and restore cellular homeostasis in vulnerable neurons. In addition, error-restrictive ribosomes probably would be able to suppress the toxic effects of proteinopathies, including those induced by polyglutamine expansions, by reducing the misincorporation of erroneous amino acids into already aggregation-prone sequences (Tenchov et al., 2024).

However, implementing error-restrictive ribosomes as a therapy in humans presents several technical and biological challenges. One consideration is the balance between translation speed and accuracy. Excessively stringent decoding could slow protein synthesis, impairing cell viability, as shown in many studies in bacteria (Johansson et al., 2012; Agarwal et al., 2015). While the *RPS23* K60R mutation demonstrates that moderate fidelity enhancement in higher eukaryotes (not mammals) can be achieved without significant fitness costs (Martinez-Miguel et al., 2021), further studies are needed to define the fitness cost or other potential negative effects of error-restrictive translation in mammalian systems.

Tissue-specific delivery in adult (or even aged) patients represents a more severe technical problem. Unlike model organisms, where germline editing is feasible, therapeutic application in humans would require expression of mutant ribosomal proteins in vulnerable neuronal populations (primarily in the brain). Current advances in gene therapy, including adeno-associated virus vectors or mRNA-based delivery systems, offer potential approaches for such targeted intervention, but application of such techniques in preventative therapy for NDDs is likely far away.

Finally, the long-term effects of expressing modified ribosomes must be assessed. While neurons are post-mitotic cells that may particularly benefit from enhanced fidelity (Bottger et al., 2025), potential off-target effects in proliferative tissues must be minimized. Tissue-specific promoters and regulated expression systems may help mitigate such risks.

### Potential of pharmacology

Another approach would be to enhance translational accuracy pharmacologically using small molecule drugs. This approach has the important advantage of temporal control, allowing treatment initiation in symptomatic individuals, and removing the drug in case side-effects occur. The first attempt to identify small molecules capable of replicating the effect of error-restrictive mutations pharmacologically has been done by Martinez-Miguel et al. (2021) and resulted in the demonstration of the potential ability of 3 drug compounds with previously described anti-aging effects to increase translation fidelity. The exact mechanism of action remains unclear; it may involve modulation of the ribosome structure, translation factor activity, or tRNA-ribosome interactions. However, the fact that known anti-aging agents also lead to improved translational fidelity should inspire further efforts to identify small molecules capable of doing this.

Several classes of small molecules have shown potential to modulate translation fidelity:

*a. Compounds targeting the ribosome*

Selective ribosome modulators can influence decoding fidelity. For example, aminoglycosides such as paromomycin and geneticin bind to the ribosome and induce stop codon readthrough by relaxing ribosomal decoding stringency, a property exploited to treat nonsense mutation genetic diseases (Nudelman et al., 2006; Zingman et al., 2007). Conversely, screening efforts may identify analogs and derivatives that interact with the ribosome and increase stringency rather than relaxing it.

Another way to adapt ribosomal function would be to identify molecules that can change or alter post-translational modifications of ribosomal proteins. Modification of post-translational modifications or their installation pathways are known with ribosomal proteins RPS23 or RPS6 in yeast (Loenarz et al., 2014) and the existence of similar mechanism(s) in mammals is postulated (Singleton et al., 2014).

*b. eEF1A inhibitors and/or modulators*

Eukaryotic elongation factor 1A (eEF1A) plays a central role in decoding by delivering aminoacyl-tRNAs to the ribosomal A-site. Its activity directly influences translation error rates; thus, targeting eEF1A may represent a viable strategy to modulate ribosomal fidelity. Compounds such as plitidepsin, which target eEF1A (Muñoz-Alonso et al., 2009), could potentially be chemically modified to enhance selection stringency during decoding, thereby reducing near-cognate tRNA incorporation. Such eEF1A-targeting derivatives, specifically selected for their ability to modulate translation fidelity, can subsequently be evaluated as therapeutic candidates for age-related NDDs. A potential limitation, however, is that plitidepsin (also known as dehydrodidemnin B) is a cyclic depsipeptide isolated from the ascidian *Aplidium albicans*, implying that its chemical modification may present substantial synthetic challenges.

*c. ISR modulators, translation reprogramming, and potential for combined therapies*

The ISR orchestrates translational reprogramming under proteotoxic stress by phosphorylating eIF2α, which attenuates global protein synthesis. Pharmacological ISR modulators such as ISRIB (ISR Inhibitor) restore translation by enhancing eIF2B function. Interestingly, ISRIB improves cognitive function in aged mice and models of traumatic brain injury and prion disease (Zhu et al., 2019).

Although ISRIB’s effect is indirect, its ability to reset translation homeostasis and reduce stress granule formation suggests potential synergy with fidelity-enhancing strategies. Combination therapies could restore proteostasis from both ends, reducing translation errors and improving the ability of cells to manage proteotoxic stress. In this way, translation fidelity-enhancing interventions may be synergized with other proteostasis-restoring therapies, such as autophagy inducers or UPR modulators, to clear existing aggregates while preventing the formation of new ones.

Taken together, strategies to enhance translation fidelity, whether through genetic modifications of ribosomal proteins or pharmacological modulation of the translational machinery, hold promise for delaying or mitigating neurodegenerative diseases. While significant technical challenges remain, especially for gene therapy, the identification of small molecules that improve translational accuracy provides a promising, controllable alternative. Future research should focus on refining these approaches and exploring combination therapies to restore proteostasis and protect neuronal health.

## Conclusion

**Figure 5:**
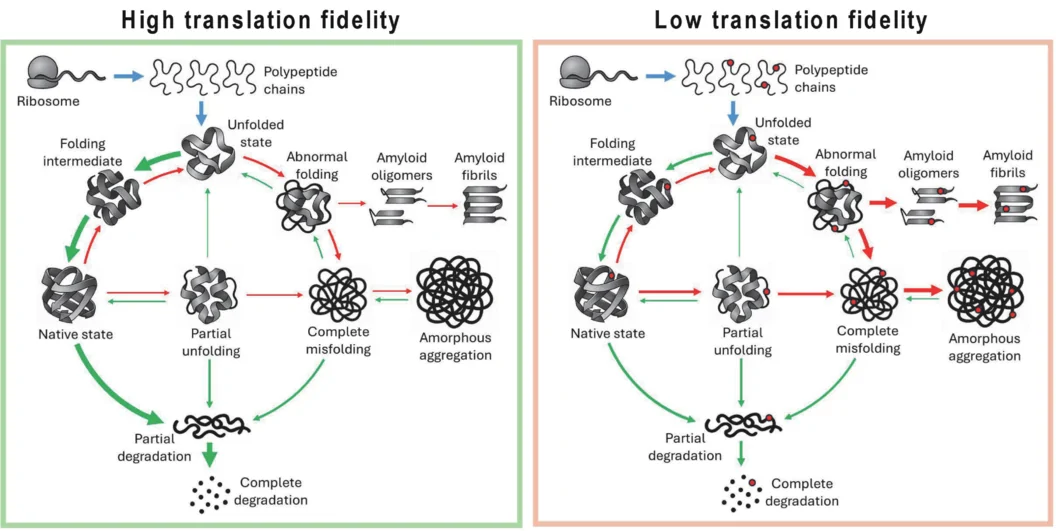
The proteostasis network functioning at normal translation fidelity (left panel) or low translation fidelity (right panel). (Left) The proteostasis network effectively supports healthy protein turnover (green arrows), controls the functional levels of proteins in their native state, and minimizes harmful off-pathway reactions, leading to the formation of unfolded proteins and aggregates (red arrows). (Right) The proteostasis network overloaded with mistranslated proteins that cannot be properly folded or easily lose their native conformation. Folding intermediates and misfolded states accumulate over time, resulting in the formation of various aggregate species, including amorphous aggregates, oligomers, and amyloid fibrils, the latter of which may be highly stable and principally non-refoldable. Adapted from Hipp et al. (2019).

Error-prone translation represents a previously underappreciated source of proteotoxic stress in NDDs. Translational errors, once considered minor contributors, are now emerging as central players in age-related proteostasis failure and neurodegeneration. With aging, the balance between translational fidelity and proteostasis is broken, resulting in the accumulation of misfolded, malfunctional, aggregation-prone proteins that evade or overwhelm degradation pathways (**Figure 5**). This molecular cascade links environmental stressors, genetic risk factors, and age-related cellular decline in translation fidelity to the pathogenesis of neurological disorders such as AD and PD. Protecting neurons by enhancing translational fidelity and restoring proteostasis is a novel approach to treating NDDs that we believe has great potential. Drug discovery efforts will not be easy since enhancing function is harder than inhibiting it. But the paradigm-shifting success of molecules that restore defective proteins and rescue protein synthesis in cystic fibrosis (Lopes-Pacheco, 2020) patients should serve as a guiding light that such efforts can be successful. Given the enormous and growing unmet medical need in NDDs, we must not shy away from this problem.

## Search Strategy

The search strategy involved brainstorming keywords and synonyms specific for the topic and combining them using Boolean operators (AND, OR) for a comprehensive search. Databases PubMed (major source), Google Scholar, or Web of Science were used. Results were narrowed by filtering for publication date, language, or article type (e.g., review). Once a list of key articles was identified, their reference lists were explored for earlier relevant works (backward searching) or for newer papers which have cited them (forward searching).

***Additional file:*** *Open peer review report 1.*

OPEN PEER REVIEW REPORT 1

## Data Availability

*Not applicable.*
