# Supplementary material for: Error-prone translation as a driver of proteostasis collapse and neurodegeneration
Source: Neural Regen Res. 2025 Dec 30;21(10):4594–602. doi: 10.4103/NRR.NRR-D-25-00795 (PMC13568678; doi:10.4103/NRR.NRR-D-25-00795)
Supplement: Supplementary file 1 [file NRR-21-4594_Suppl1.pdf]

OPEN PEER REVIEW REPORT 1

Name of journal: Neural Regeneration Research
Manuscript NO: NRR-D-25-00795
Title: Error-prone translation as a driver of proteostasis collapse and neurodegeneration: mechanistic insights into age-related neurodegenerative diseases
Reviewer's Name: Sebastian Iben
Reviewer's country: Germany

COMMENTS TO AUTHORS

Overall evaluation on article quality: Review of the manuscript titled: Error-prone translation as a driver of proteostasis collapse and neurodegeneration: mechanistic insights into age-related neurodegenerative diseases. This review provides a detailed overview about the current state of publication that discusses ribosomal errors as a driver of age-associated disease. Starting with compiling evidence that age-dependent neurodegenerative diseases are proteinopathies caused by proteostasis failure, the authors describe that there are evidences linking proteostasis failure to translational fidelity. Cytosolic translation is characterized and the different steps that can cause translational errors of different types are specified. The declining proteostasis balance due to a fading folding, maintenance and degradation capacity with aging is listed, the error-catastrophe theory of aging is not missing and the current findings after manipulation of ribosomal fidelity in animals are listed. Then the authors return to neurodegenerative diseases of the aging body and reflect on current evidences linking translational errors to these diseases of proteostasis failure. In the last paragraph the authors discuss possible intervention strategies to improve the quality of protein synthesis and then conclude that error-prone translation "represents a previously underappreciated source of proteotoxic stress"

The manuscript is very well written and spans a broad but sufficient detailed overview about a topic that is of utmost relevance for our understanding of neurodegenerative diseases but overlooked by the mainstream research. Therefor the manuscript should be published for to provoke discussion and provide more attention for the here presented hypotheses. However the reviewer recommends some careful revisions.

1. In the manuscript there is the assumption that an age-dependent decline in translational fidelity, as recently been demonstrated for mice is also involved in the pathogenesis of neurodegeneration in humans. As even in different rodent species the accuracy of the translation co-evolved with longevity (Ke et al, 2017) and thus differs, it is not self-evident that humans did not develop strategies to maintain translational fidelity especially in the brain, as human lifespan exceeds that of mice by 20 fold. As the authors correctly state, the studies about human aging fibroblasts were conducted with insensitive assays and long ago. We are still ignorant about ribosomal error-rate in human aging. And it is obvious that we need here more research. I would recommend to include these white spaces in knowledge into the manuscript that focusses more on insights gained in animal models and less in human disease. This is inasmuch important as often the animal models do not recapitulate central features of the neurodegenerative diseases, eg they do not develop neurodegeneration. "mice do not get Alzheimer" (Behl, C Alzheimer disease research 2022).
2. Human diseases with translational fidelity alterations like dyskeratosis congenita, Diamond Blackfan anemia and the even more topically relevant, the premature-aging diseases Cockayne syndrome and trichothiodystrophy identified by the Iben group are not mentioned. To the reviewers knowledge there are only few groups working on the specific hypothesis that ribosomal errors provoke neurodegeneration and a review discussing this topic needs to cite the relevant literature accordingly.

The publications of the Iben group describe the pathogenesis of premature aging diseases from RNA polymerase I transcription disturbances to translational infidelity and neurodegeneration and need to be cited accordingly. Moreover, the recent publication by Wagner et al 2024 is linking ribosomal translational infidelity to Huntington`s disease, an age-related neurodegenerative disease, and is thus highly relevant for the review. Please read and cite this literature.
